# Effectiveness of Reducing Craving in Alcohol Use Disorder Using a Serious Game (SALIENCE): Randomized Controlled Trial

**DOI:** 10.2196/42194

**Published:** 2023-11-07

**Authors:** Antonia Weber, Yury Shevchenko, Sarah Gerhardt, Sabine Hoffmann, Falk Kiefer, Sabine Vollstädt-Klein

**Affiliations:** 1 Department of Addictive Behavior and Addiction Medicine Central Institute of Mental Health Medical Faculty Mannheim, Heidelberg University Mannheim Germany; 2 Research Methods, Assessment, and iScience Department of Psychology University of Konstanz Konstanz Germany; 3 Mannheim Center for Translational Neurosciences (MCTN) Medical Faculty Mannheim Heidelberg University Mannheim Germany; 4 Feuerlein Center on Translational Addiction Medicine Heidelberg University Heidelberg Germany

**Keywords:** alcohol approach bias, alcohol attentional bias, alcohol use disorder, alcohol, attention, cognitive bias modification therapy, craving, cue-exposure therapy, decision-making training, decision-making, incentive salience, serious games, therapy, training

## Abstract

**Background:**

Alcohol use disorder (AUD) has become a major global health problem. Therapy for this condition is still a great challenge. Recently, it has become increasingly evident that computer-based training is a valuable addition to the treatment of addictive disorders.

**Objective:**

This study aims to evaluate the web-based serious game SALIENCE (Stop Alcohol in Everyday Life-New Choices and Evaluations) as an add-on therapy for AUD. It combines the cue-exposure therapy approach with elements of decision-making training, enhanced by interactive panoramic images. The effects of SALIENCE training on levels of craving, attention, and cognitive bias are investigated.

**Methods:**

In a randomized controlled trial, 62 participants with AUD undergoing 3 weeks of an extended alcohol detoxification program were randomly allocated to an intervention and a control group. A total of 49 individuals (mean age 44.04 y; 17/49, 35% female) completed all sessions and were included in the analysis. Only pretreatment data were available from the other 13 patients. Participants answered questionnaires related to alcohol consumption and craving and completed neuropsychological tasks at the beginning of the study and 2 weeks later to evaluate levels of attention and cognitive biases. During the 2-week period, 27 of the participants additionally performed the SALIENCE training for 30 minutes 3 times a week, for a total of 6 sessions.

**Results:**

We observed a significant decrease in craving in both groups: the control group (mean 15.59, SD 8.02 on the first examination day vs mean 13.18, SD 8.38 on the second examination day) and the intervention group (mean 15.19, SD 6.71 on the first examination day vs mean 13.30, SD 8.47 on the second examination day; *F*_1,47_=4.31; *P*=.04), whereas the interaction effect was not statistically significant (*F*_1,47_=0.06; *P*=.80). Results of the multiple linear regression controlling for individual differences between participants indicated a significantly greater decrease in craving (*β*=4.12; *t*_36_=2.34; *P*=.03) with the SALIENCE intervention. Participants with lower drinking in negative situations reduced their craving (*β*=.38; *t*_36_=3.01; *P*=.005) more than people with higher drinking in negative situations.

**Conclusions:**

The general effectiveness of SALIENCE training as an add-on therapy in reducing alcohol craving was not confirmed. Nevertheless, taking into account individual differences (gender, duration of dependence, stress, anxiety, and drinking behavior in different situations), it was shown that SALIENCE training resulted in a larger reduction in craving than without. Notably, individuals who rarely consume alcohol due to negative affect profited the most from SALIENCE training. In addition to the beneficial effect of SALIENCE training, these findings highlight the relevance of individualized therapy for AUD, adapted to personal circumstances such as drinking motivation.

**Trial Registration:**

ClinicalTrials.gov NCT03765476; https://clinicaltrials.gov/show/NCT03765476

## Introduction

With a per capita consumption of 13.4 liters of pure ethanol per year, Germany’s alcohol consumption is higher than the average of 9.8 liters for European member states. It is predicted that alcohol consumption will continue to increase worldwide. More than 200 kinds of diseases are associated with risky alcohol consumption, which makes it 1 of the 5 major risk factors for diseases, impairments, and deaths worldwide. Overconsumption of alcohol led to approximately 3 million deaths in 2016, accounting for 5.3% of all deaths globally. In Germany, 6.8% of the population have alcohol use disorder (AUD) [1], but only 17% of individuals with AUD undergo therapy. Without further interventions, 85% of patients relapse after treatment [2,3]. These statistics highlight the difficulty of treating AUD and the need for further work to improve the effectiveness of therapy.

Craving is an important factor that contributes to relapse [4] and is considered one of the diagnostic criteria for alcohol dependence [5,6]. Craving correlates positively with the amount of alcohol consumed and can serve as a measure of relapse likelihood [7-9]. Both pharmacological and psychotherapeutic treatments are used to reduce craving and thereby prevent relapse [10-12].

Craving is triggered, among other reasons, by addiction-associated stimuli. Individuals with AUD are more sensitive to alcohol-associated stimuli than healthy individuals [13,14]. This means that the presentation of alcohol-associated stimuli, referred to as cue exposure, elicits higher cue reactivity in individuals with alcohol dependence. The cue reactivity refers to various physiological reactions, such as an increased heart rate, an increased pulse, an increase in electrodermal conductivity, a change in cerebral blood flow, and the release of certain neurotransmitters [15-20]. At the psychological level, cue reactivity manifests itself as addictive craving and changes in mood [19]. Consequently, cue reactivity is a significant factor that subconsciously influences behavior and increases the risk of relapse [21]. As alcohol-associated stimuli are difficult to avoid in everyday life, they represent a major challenge for addicts in the pursuit of abstinence.

The incentive-sensitization theory of addiction describes why certain stimuli trigger a strong craving for alcohol even without the presence of withdrawal symptoms [22]. According to this theory, enduring alcohol consumption causes persistent neuroadaptation. Stimuli that are usually followed by the consumption of alcohol gain importance and attractiveness due to increasing pathological hypersensitization in the mesolimbic dopaminergic system. Thus, these cue stimuli are continually linked to the reward that follows them through the effect of alcohol. Hence the expectation is created that a positive consequence always follows alcohol-associated stimuli. Because of this sensitization process, the presentation of the stimulus releases dopamine and triggers a craving for alcohol. The acquired motivational significance of alcohol-associated stimuli is referred to as “incentive salience” [22,23].

The incentive salience leads to an unconscious shift of attention toward substance-associated stimuli, such as social context, sounds, smells, and visual impressions, which is called attentional bias. As a result, substance-associated stimuli attract more attention than neutral stimuli. Moreover, individuals with AUD tend to approach substance-associated stimuli automatically and subconsciously before this impulse can be reconsidered or reflected upon [24,25]. Therefore, an imbalance between impulsive and deliberate decisions is characteristic of pathological decision-making in individuals with AUD [26].

Previous studies have found that cognitive biases such as alcohol approach bias and alcohol attentional bias can be reduced through computer-assisted training [27,28]. Cognitive Bias Modification Therapy (CBMT) is a nondrug psychotherapeutic approach aimed at improving cognitive impairments. It attempts to replace dysfunctional cognitive processes that lead to undesirable actions with alternative processing through systematic, repeated exercises [29].

Moreover, cue-exposure therapy (CET) can reduce craving for alcohol in the long term, thereby reducing the risk of relapse [18,30,31]. CET is an approach where a learned response is triggered by the repeated presentation of conditioned stimuli [32], which causes a systematic desensitization by not reinforcing the alcohol-associated stimuli. Within the framework of extinction learning, the link between the pleasurable effects of alcohol consumption and the conditioned stimulus is gradually disconnected [31]. The psychophysiological response toward it is gradually diminished, thus modulating misguided consumption behavior [33-35].

A feasibility study with 32 patients with alcohol dependence has shown that the low-cost and easy-to-implement add-on CBMT SALIENCE (Stop Alcohol in Everyday Life-New Choices and Evaluations) with elements of CET was very well received by the participants. Regardless of each participant’s individual experience with computers, they all rated the application as user-friendly and realistic. The participants indicated that they were willing to incorporate the training into their daily lives [36].

The goal of this randomized controlled study is therefore to investigate the effects of the serious game SALIENCE as a low-cost and easy-to-implement add-on therapy on alcohol craving, attention, and cognitive bias. We aim to provide a more detailed understanding of how individual differences affect the efficiency of this add-on therapy. By adding 9 new scenarios to the SALIENCE training [36], this study will also test an extended version of the add-on therapy with 16 scenarios embedded in 3 different stories.

We expected that the computer-based SALIENCE training as an add-on to standard therapy would reduce craving, increase attention and concentration performance, and decrease alcohol approach and alcohol attentional biases in comparison with standard therapy only.

## Methods

### Overview

We conducted a parallel randomized controlled study at the Department of Addictive Behavior and Addiction Medicine, Central Institute of Mental Health in Mannheim, Germany. For more detailed information, see the CONSORT-EHEALTH checklist (Multimedia Appendix 1).

### Participants

A total of 62 participants were recruited for the study. Randomly, 31 participants were assigned to the SALIENCE computer training intervention group and 31 to the control group, which received standard therapy in addition to the computer training.

All participants were aged between 18 and 65 years and had AUD according to the Diagnostic and Statistical Manual of Mental Disorders, 5th Edition (DSM-5) [5]. They had been abstinent for at least 72 hours and a maximum of 6 weeks. The participants did not have an uncorrectable visual impairment and were able to communicate sufficiently with the investigator in written and verbal form. Exclusion criteria were severe withdrawal symptoms quantified by the Clinical Institute Withdrawal Assessment for Alcohol (CIWA-Ar) questionnaire [37], alcohol intoxication, and use of pharmacotherapy with withdrawal-suppressing substances within the past 3 days. Severe internal, neurological, and psychiatric comorbidities as well as axis I disorders within the past 12 months also led to exclusion. To obtain a more representative sample, participants with the following disorders were eligible to take part in the study: substance use disorders, anxiety disorders and phobias, mild or moderate depressive episodes, somatoform disorders, adjustment disorders, sleep disorders, and eating disorders.

After removing the data of 13 participants who did not complete the study (9 participants in the control group and 4 participants in the intervention group), the data of 49 participants (17 female and 32 male individuals with a mean age of 44, SD 12 y) were used for the analysis. The participants were individuals with AUD who were undergoing an extended detoxification treatment. Among these participants, 25 were undergoing partial inpatient treatment, and 24 were undergoing inpatient treatment.

### Design

**Figure 1:**
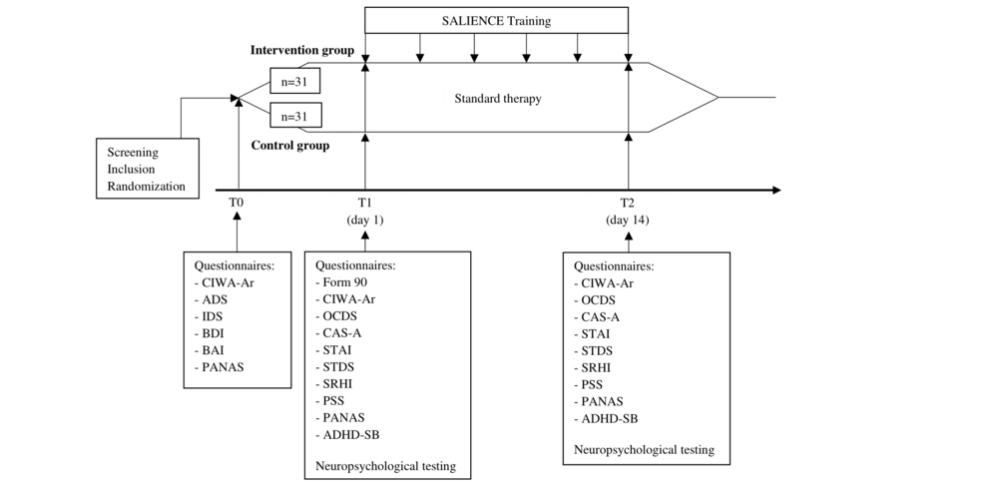
Flowchart of the study design (intervention group: n=27; control group: n=22). Only the intervention group completed the additional SALIENCE (Stop Alcohol in Everyday Life-New Choices and Evaluations) training. ADHD-SB: Attention-Deficit/Hyperactivity Disorder Self-Assessment Scale; ADS: Alcohol Dependence Scale; BAI: Beck Anxiety Inventory; BDI: Beck Depression Inventory; CAS-A: Craving Automated Scale for Alcohol; CIWA-Ar: Clinical Institute Withdrawal Assessment for Alcohol; IDS: Inventory of Drinking Situations; OCDS: Obsessive Compulsive Drinking Scale; PANAS: Positive and Negative Affect Schedule; PSS: Perceived Stress Scale; SRHI: Self-Report Habit Index; STAI: State-Trait Anxiety Inventory; STDS: State-Trait Depression Scale.

Each participant took part in 1 screening day (T0) before being randomly allocated to a group, followed by 2 examination days with a 2-week interval. In addition, the intervention group attended the computer-based SALIENCE training 3 times a week between the first examination day (T1) and the second examination day (T2). Thus, a total of 6 training sessions were completed. Eligibility for participation was assessed on T0. Participants were asked for sociodemographic data, and baseline psychometric data were collected using questionnaires. At T1, previous drinking behavior was recorded, and other questionnaires were filled out. Furthermore, 4 neuropsychological tests were carried out. The measurements at T2 were conducted 2 weeks later in the same way as at T1, except for the drinking quantity. In the meantime, the extended detoxification treatment was continued for both groups (Figure 1).

### Framework of SALIENCE

The name SALIENCE stands for “Stop Alcohol in Everyday Life-New Choices and Evaluations” and refers to a pathological level of incentive salience. The program combines training elements of CET and decision-making using interactive panoramic images. The training program was designed as a lightweight web-based application and does not require any additional software. Thus, it can be used on different devices, such as computers, laptops, and tablets. The game management system allows users to expand and customize scenarios without much additional programming.

Researchers or therapists can add and modify addiction-associated photos (eg, pubs and liquor stores) in the browser-based application through a simple interface. In this way, the game simulates, as realistically as possible, the expected future risk situations to which a patient may be exposed. In addition, a habituation effect can be counteracted by being able to easily edit the scenarios.

The basic framework of the program was created as part of a master’s thesis (Ueberle C, unpublished data, 2015). Within the scope of a bachelor’s thesis, the program was later complemented by the game management system (Weigand T, unpublished data, 2016).

**Figure 2:**
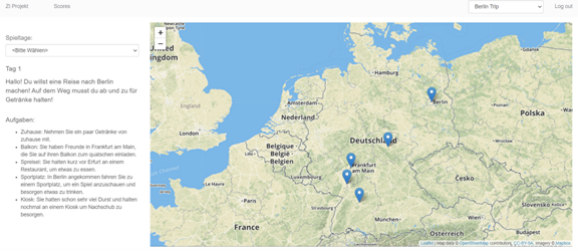
Screenshot of the story interface in SALIENCE (Stop Alcohol in Everyday Life-New Choices and Evaluations). The map of Germany is presented in this example. Players can select any location on the map to start a game.

**Figure 3:**
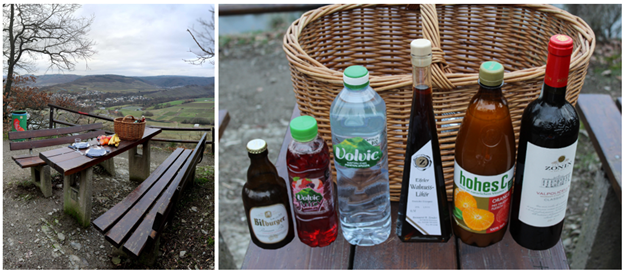
Screenshot of the game interface in SALIENCE (Stop Alcohol in Everyday Life-New Choices and Evaluations). The picnic scenario is presented in this example. Players have to click on all nonalcoholic drinks as quickly as possible and avoid clicking on alcoholic drinks.

In this serious game, the players embark on a web-based journey through Germany, embedded in a story. Along the way, the participants are confronted with scenarios where alcohol is usually consumed, which could also increase their craving in real life (eg, a visit to a bar, a family party, and a walk to the kiosk). In combination with the descriptions, the situations are highly realistic and simulate critical moments when addictive cravings and relapses can occur. There is always 1 map [38] per story with various locations on it (Figure 2). Each location holds a minigame, which players can enter by clicking on it. The game presents the player with a real-world panoramic photo where alcoholic and nonalcoholic beverages can be seen (Figure 3). To treat pathological decision-making, patients undergo a decision training in which they have to choose nonalcoholic beverages over alcoholic ones as fast as possible. Severe side effects of the SALIENCE training are unlikely since it is a web-based exposure.

In this study, a training session consisted of 3 different stories, each with 5 to 6 associated locations, or minigames. With a duration of about 30 minutes, a training session can be integrated into everyday life without much effort.

### Questionnaires

To collect addiction-specific baseline data, the CIWA-Ar [37], the Alcohol Dependence Scale (ADS) [39], and the Inventory of Drinking Situations (IDS) [40] were applied at T0. At T1, the Beck Depression Inventory (BDI) [41], the Beck Anxiety Inventory (BAI) [42], the Positive and Negative Affect Schedule (PANAS) [43], and the Attention-Deficit/Hyperactivity Disorder Self-Assessment Scale (ADHD-SB) [44] were administered. Using the Form 90 questionnaire, the individual alcohol consumption of each participant was recorded at T1 [45]. Further questionnaires were completed at T1 and T2; the CIWA-Ar was used to record withdrawal symptoms. The Obsessive Compulsive Drinking Scale (OCDS) [46] was used to measure the severity of craving in each individual. In addition to the OCDS sum score, the subscales “thoughts” and “actions” were used in the analysis. The Craving Automated Scale for Alcohol (CAS-A) [47] captured and estimated habitual and automated alcohol consumption. The State-Trait Anxiety Inventory (STAI) [48] measured the level of anxiety. The State-Trait Depression Scale (STDS) [49] provided information on the expression of state or trait characteristics of the individual’s depressive symptoms. The Self-Report Habit Index (SRHI) [50] quantified different facets of drinking habits. The Perceived Stress Scale (PSS) [51] mapped the participants’ perception of stress.

In addition, 3 questions about craving were administered at T1 and T2, using a numeric rating scale from 0 to 100. The 3 questions were:

- During the last 7 days, how strong was your craving for alcohol (the desire for alcohol while not drinking) on average?
- Please think back once within the last 7 days to the moment when the craving for alcohol was strongest. How strong was this craving?
- During the last 7 days, how often did you have cravings for alcohol (the desire for alcohol while not drinking)?

The average of the responses to these questions was used as a separate variable in the analysis and referred to as “strength and frequency of craving.”

### Neuropsychological Tests

The test battery included 4 neuropsychological tests, which were performed at T1 and T2 at the very beginning of each session.

The d2 Attention Test evaluated the individuals’ attention and concentration performance [52]. The number of correct and incorrect responses was used for the analysis.

The Approach Avoidance Task (AAT) [53] measured the participants’ alcohol approach bias. As dependent variables, we used the number of correct responses and the mean reaction time for alcoholic and nonalcoholic beverages. As a measure of the bias, we calculated the difference between reaction times for correctly selected alcoholic and nonalcoholic beverages.

The Dot Probe Task [54] and the Alcohol Stroop Task [55,56] assessed alcohol attentional bias. For the Dot Probe Task, we used the number of correct responses and the mean reaction time in different conditions. Moreover, we used the difference in reaction times between congruent and incongruent stimuli to compute the Dot Probe Score, which is a measure of the bias. For the Alcohol Stroop Task, we calculated the difference in reaction times between alcohol and household items.

### Statistical Analysis

We used SPSS software (version 27.0, SPSS Inc) to perform statistical analyses. The significance level was set to .05. First, we analyzed the baseline differences between the intervention and control groups using independent 2-sample 2-tailed *t* tests and chi-square tests. Using repeated measures ANOVA, we evaluated possible dissimilarities between the intervention and control groups across the study period. In this way, the scores for craving, attention performance, alcohol attentional bias, and alcohol approach bias were examined more closely. Time was set as a within-subject factor, and group condition was set as the between-subjects factor. In addition to the confirmatory analyses, exploratory analyses were conducted using the R software (version 4.0.2; R Foundation for Statistical Computing). We conducted 2 multiple linear regression analyses to evaluate the effect of the SALIENCE training on the changes in craving score and alcohol approach bias. Gender, duration of dependency, PSS, STAI, and IDS scores were added as predictors to the models. To examine the moderator effect of the addiction type, the interaction of the “negative situations” subscale of the IDS and participation in the training were included in the models.

### Ethical Considerations

The study protocol was planned and conducted in accordance with the Declaration of Helsinki (registration at ClinicalTrials.gov; NCT03765476). The responsible ethics committee (Committee II of the University of Heidelberg, file 2018-593N-MA) approved the study. Trial administrators obtained fully informed written consent from each participant before taking part in the study. The data was de-identified and participants were not compensated for their participation.

## Results

### Descriptive Analysis

**Figure 4:**
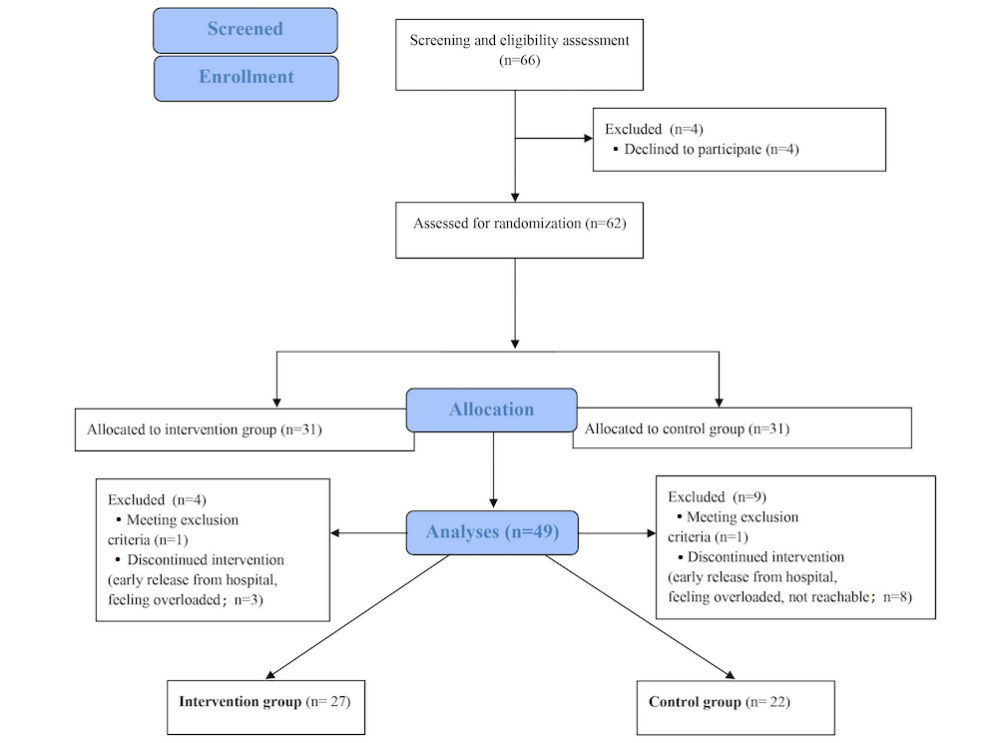
Consolidated Standards of Reporting Trials (CONSORT) flowchart.

We included 49 participants (intervention group: n=27, 55%; control group: n=22, 45%) in our analyses (Figure 4 [57]). Intervention and control groups differed significantly in age (*P*=.03), and duration of dependence (*P*=.004) at baseline. While the intervention group was younger (intervention group: mean 40.56, SD 11.59 y; control group: mean 48.32, SD 12.18 y), they also had a shorter duration of dependence than the control group (intervention group: mean 25.70, SD 8.66 y; control group: mean 36.09, SD 13.87 y). The remaining sociodemographic and psychometric measures did not differ significantly (all *P* values >.05; Table 1).

**Table 1:** Sociodemographic variables and questionnaire scores at baseline.

Variables	Intervention group (n=27)	Control group (n=22)	*t* test (*df*)^a^	Chi-square (*df*)^a^	*P* value
Age (y), mean (SD)	40.56 (11.59)	48.32 (12.18)	–2.27 (47)	N/A^b^	.03
Female, n (%)	6 (22)	11 (50)	N/A	2.99 (1)^c^	.08
Married, n (%)	4 (15)	5 (23)	N/A	2.44 (3)	.49
Duration of dependence (y), mean (SD)	25.70 (8.66)	36.09 (13.87)	–3.06 (34)	N/A	.004
Previous inpatient detoxification, n (%)	13 (48)	10 (45)	N/A	0.0 (1)	>.99
Psychological comorbidities, n (%)	15 (56)	12 (54)	N/A	0.0 (1)	>.99
ADS^d^ sum score, mean (SD)	18.69 (7.70)	16.14 (9.25)	1.03 (46)	N/A	.31
IDS100^e^ positive situations, mean (SD)	12.99 (7.07)	12.11 (7.29)	0.42 (46)	N/A	.68
IDS100 negative situations, mean (SD)	11.88 (5.03)	11.12 (6.29)	0.46 (46)	N/A	.65
BDI^f^ sum score, mean (SD)	18.33 (11.82)	13.95 (12.52)	1.25 (47)	N/A	.22
BAI^g^ sum score, mean (SD)	16.53 (12.77)	13.18 (14.51)	0.84 (46)	N/A	.41
PANAS^h^ positive affect trait, mean (SD)	25.15 (7.78)	26.09 (10.93)	–0.34 (47)	N/A	.74
PANAS negative affect trait, mean (SD)	27.52 (7.03)	22.68 (9.76)	1.95 (47)	N/A	.06
ADHD^i^ sum score, mean (SD)	18.11 (9.23)	14.73 (11.70)	1.11 (47)	N/A	.28
F90^j^ consumption of alcohol (g), mean (SD)	9573.31 (7979.91)	11395.68 (7827.36)	–0.80 (46)	N/A	.43
F90 drinks per day, mean (SD)	8.86 (7.39)	10.55 (7.25)	–0.80 (46)	N/A	.43
F90 drinks per drinking day, mean (SD)	15.75 (9.11)	15.79 (9.23)	–0.02 (46)	N/A	.99
F90 drinks per heavy drinking day, mean (SD)	25.91 (29.80)	18.88 (14.05)	1.20 (46)	N/A	.29
F90 percent days abstinent, mean (SD)	44.10 (28.36)	33.94 (23.84)	1.35 (46)	N/A	.18
F90 percent drinking days, mean (SD)	55.90 (28.36)	66.57 (23.45)	–1.43 (46)	N/A	.16
F90 percent heavy drinking days, mean (SD)	49.83 (31.42)	62.37 (26.59)	–1.50 (46)	N/A	.14

^a^We performed the Welch *t* test. Some participants had missing values, which led to different degrees of freedom.

^b^N/A: not applicable.

^c^Pearson chi-square test with Yates continuity correction.

^d^ADS: Alcohol Dependence Scale.

^e^IDS100: Inventory of Drinking Situations.

^f^BDI: Beck Depression Inventory.

^g^BAI: Beck Anxiety Inventory.

^h^PANAS: Positive and Negative Affect Schedule.

^i^ADHD: attention-deficit/hyperactivity disorder.

^j^F90: Form 90.

### Craving

Using repeated measures ANOVA, we compared the craving scores at T1 and T2 in the control and intervention groups. We observed a significant decrease in the craving OCDS total sum score (*F*_1,47_=4.31; *P*=.04) and the OCDS “actions” subscale (*F*_1,47_=8.21; *P*=.01) over the study period in both groups. For the OCDS “thoughts” subscale (*F*_1, 47_=0.55; *P*=.46) and “craving intensity and frequency” scale (*F*_1, 47_=3.35; *P*=.07), the decrease over time was not statistically significant. Neither the OCDS total sum score (*F*_1,47_=0.06; *P*=.80), the OCDS “thoughts” subscale (*F*_1,47_=0.05; *P*=.83), the OCDS “actions” subscale (*F*_1,47_=0.05; *P*=.82), nor the “strength and frequency of craving” scale (*F*_1,47_=0.19; *P*=.67) had significant time-group interactions (Table 2).

**Table 2:** Obsessive Compulsive Drinking Scale scores.

Variables	Intervention group (n=27)		Control group (n=22)		Effect of time test		Time-group interaction test	
	T1^a^, mean (SD)	T2^b^, mean (SD)	T1, mean (SD)	T2, mean (SD)	*F* test (*df*)	*P* value	*F* test (*df*)	*P* value
Total sum score	15.19 (6.71)	13.30 (8.47)	15.59 (8.02)	13.18 (8.38)	4.31 (1, 47)	.04	0.06 (1, 47)	.80
Subscale “thoughts”	5.22 (3.77)	4.93 (4.41)	5.64 (4.47)	5.01 (4.56)	0.55 (1, 47)	.46	0.05 (1, 47)	.83
Subscale “actions”	9.96 (4.19)	8.37 (4.58)	9.95 (4.11)	8.09 (4.25)	8.21 (1, 47)	.01	0.05 (1, 47)	.82
Strength and frequency of craving	4.69 (3.13)	4.22 (3.22)	3.65 (3.01)	2.89 (2.74)	3.35 (1, 47)	.07	0.19 (1, 47)	.67

^a^T1: first examination day.

^b^T2: second examination day.

### Attention

The number of correct and incorrect responses in the d2 Attention Test was computed for each participant. We conducted repeated measures ANOVA to compare the outcomes at T1 and T2 in the control and intervention groups. Over the 2 measurements, all participants improved their task performance, as indicated by a significant increase in the number of correctly selected items in both groups (*F*_1,47_=79.20; *P*<.001). However, there was no statistically significant time-group interaction (*F*_1,47_=0.00; *P*=.98). The number of incorrectly selected items did not change over time in both groups (*F*_1,47_=0.40; *P*=.53) or in 1 group specifically (*F*_1,47_=1.99; *P*=.17; Table 3).

**Table 3:** d2 Attention Test scores.

Responses	Intervention group (n=27)		Control group (n=22)		Effect of time test		Time-group interaction test	
	T1^a^, mean (SD)	T2^b^, mean (SD)	T1, mean (SD)	T2, mean (SD)	*F* test (*df*)	*P* value	*F* test (*df*)	*P* value
Correctly selected	11.88 (3.60)	15.17 (4.22)	8.87 (4.40)	12.14 (4.92)	79.20 (1, 47)	<.001	0.00 (1, 47)	.98
Incorrectly clicked	0.42 (1.08)	0.48 (0.99)	0.68 (1.00)	0.52 (9.44)	0.40 (1, 47)	.53	1.99 (1, 47)	.17

^a^T1: first examination day.

^b^T2: second examination day.

### Alcohol Approach Bias

Over the 2 measures at T1 and T2, all participants became faster in correctly responding to the task stimuli, as confirmed by a significant main effect of time in repeated measures ANOVA for alcoholic (*F*_1,47_=5.11; *P*=.03) and nonalcoholic beverages (*F*_1,47_=5.03; *P*=.03). However, there was no statistically significant time-group interaction (alcohol: *F*_1,47_=0.59; *P*=.45; no alcohol: *F*_1,47_=0.43; *P*=.52). For the other variables, there were neither significant changes over time nor time-group interactions (Table 4).

**Table 4:** Approach Avoidance Task scores.

Variables	Intervention group (n=27)		Control group (n=22)		Effect of time test			Time-group interaction test	
	T1^a^, mean (SD)	T2^b^, mean (SD)	T1, mean (SD)	T2, mean (SD)	*F* test (*df*)	*P* value	*F* test (*df*)		*P* value
Number of correct alcoholic answers	19.33 (0.68)	19.11 (0.93)	18.82 (1.30)	19.00 (0.98)	0.01 (1, 47)	.92	1.01 (1, 47)		.32
Number of correct nonalcoholic answers	18.74 (1.13)	19.04 (1.09)	18.05 (2.19)	18.50 (1.17)	1.68 (1, 47)	.20	0.08 (1, 47)		.79
Average reaction time for the correct alcoholic answer (ms)	1018.98 (328.14)	927.23 (188.06)	1325.56 (627.94)	1139.12 (406.84)	5.11 (1, 47)	.03	0.59 (1, 47)		.45
Average reaction time for the correct nonalcoholic answer (ms)	1098.36 (324.10)	1014.12 (242.70)	1371.67 (597.90)	1217.73 (529.60)	5.03 (1, 47)	.03	0.43 (1, 47)		.52
Difference in reaction times for correct alcoholic and nonalcoholic answers (ms)	–79.39 (212.52)	–86.89 (97.47)	–46.11 (312.71)	–78.62 (233.52)	0.23 (1, 47)	.64	0.09 (1, 47)		.77

^a^T1: first examination day.

^b^T2: second examination day.

### Alcohol Attentional Bias

The alcohol attentional bias was assessed in the Alcohol Stroop Task and the Dot Probe Task. In the Alcohol Stroop Task, the alcohol attentional bias was measured as the difference in reaction time between alcohol and household items (eg, rum vs towel). Using repeated measures ANOVA, we found that the alcohol attentional bias did not decrease significantly over the study period (*F*_1,47_=0.01; *P*=.95). There was also no significant time-group interaction (*F*_1,47_=0.07; *P*=.80; Table S1 in Multimedia Appendix 2). However, mean reaction times in the different categories decreased.

Based on repeated measures ANOVA, no significant changes between T1 and T2 measurements were found in the Dot Probe Task. There were no relevant effects of time (*F*_1,45_=1.78; *P*=.19) nor interactions between time and groups (*F*_1,45_=0.37; *P*=.55; Table S2 in Multimedia Appendix 2).

### Exploratory Analyses

#### Overview

Although there were no significant group differences found in the repeated measures ANOVA, it may be that the participants benefited differently from the intervention, for example, depending on the type of their addiction [58]. Further exploratory analyses were conducted to determine whether certain individual differences, such as sociodemographic, behavioral, and psychometric variables, moderated the effect of the SALIENCE intervention.

#### The Effect of SALIENCE Intervention on Craving

We conducted multiple linear regression analyses to evaluate the effect of the SALIENCE intervention on craving. Gender, duration of addiction, stress (PSS), anxiety (STAI), and drinking behavior in positive and negative situations (IDS) were used as predictor variables in the model. To examine the moderator effect of addiction type on craving, we included the interaction between the level of drinking in negative situations and training in the model. As the dependent variable, we used the “strength and frequency of craving” score in the OCDS questionnaire. We calculated the difference in the craving score between 2 measurements (T2 minus T1). The negative value of the “craving difference” variable means that the craving decreased from T1 to T2.

**Figure 5:**
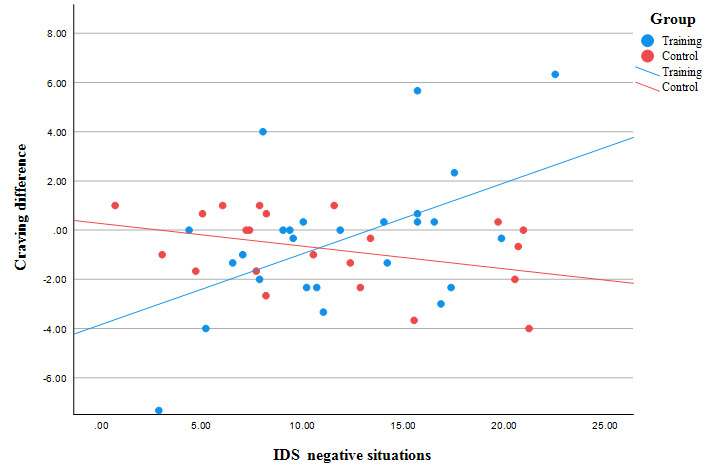
The change in craving (craving on the second examination day [T2] minus craving on the first examination day [T1]) for participants with different levels of drinking in negative situations in training and control groups. IDS: Inventory of Drinking Situations.

Results of the multiple linear regression indicated that the SALIENCE intervention significantly reduced craving in the intervention group (*β*=–4.12; *t*_36_=–2.34; *P*=.03; Table 5). The interaction of the IDS “negative situations” scale and the training intervention was statistically significant (*β*=.38; *t*_36_=3.01; *P*=.005), that is, in the intervention group, people with a lower level of drinking in negative situations reduced their craving more than people with a higher level of drinking in negative situations (Figure 5).

**Table 5:** Effect estimates for the change in craving (craving on the second examination day [T2] minus craving on the first examination day [T1]; n=46; F9,36=2.03; *P*=.06; *R*²=0.34; adjusted *R*²=0.17).

Variables	Regression coefficient (SE)	*t* value	*P* value
Intercept	1.29 (2.56)	0.51	.62
Male	0.55 (0.85)	0.64	.52
Duration of dependency	–0.01 (0.03)	–0.08	.94
PSS^a^ at T1	0.13 (0.07)	1.87	.07
STAI^b^ State at T1	0.01 (0.04)	0.09	.93
STAI Trait at T1	–0.9 (0.05)	–1.89	.07
IDS^c^ (drinking in positive situations)	–0.03 (0.06)	–0.51	.62
IDS (drinking in negative situations)	–0.03 (0.11)	–0.27	.79
Training	–4.12 (1.76)	–2.34	.02
IDS (drinking in negative situations) × training	0.38 (0.13)	3.01	.005

^a^PSS: Perceived Stress Scale.

^b^STAI: State-Trait Anxiety Inventory.

^c^IDS: Inventory of Drinking Situations.

#### The Effect of SALIENCE Intervention on Alcohol Approach Bias

We performed a multiple regression analysis to measure the effect of the SALIENCE intervention on alcohol approach bias. The dependent variable was the bias, measured as the difference in reaction times for alcohol and nonalcohol items in the AAT (calculated as “the reaction time in alcohol trials minus the reaction time in nonalcohol trials”). To calculate the change in the bias, the bias value of T1 was subtracted from T2. The variable was titled “AAT difference” and used as a dependent variable in the analysis. We set the same parameters as predictor variables as in the model with craving.

**Figure 6:**
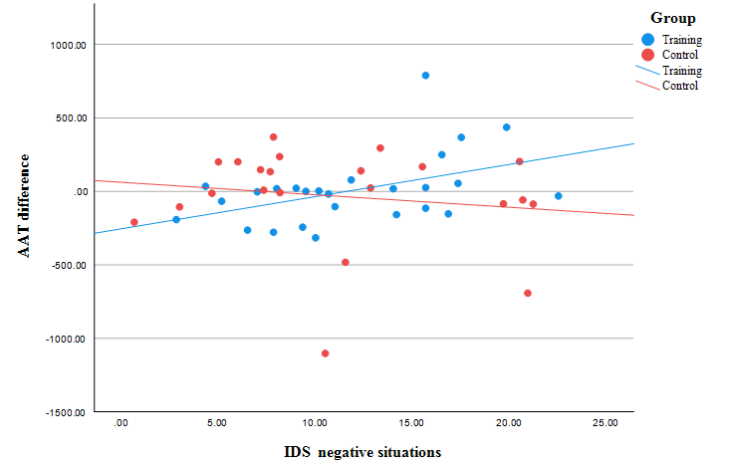
The change in the alcohol approach bias (the bias on the second examination day [T2] minus the bias on the first examination day [T1]) for participants with different levels of drinking in negative situations in training and control groups. AAT: Approach Avoidance Task; IDS: Inventory of Drinking Situations.

Results of the multiple linear regression analysis indicated that the SALIENCE intervention significantly decreased the bias (*β*=–580.81; *t*_36_=–2.62; *P*=.01; Table 6). The interaction between the IDS “negative situations” scale and training was significant (*β*=43.98; *t*_36_=2.77; *P*=.009), that is, in the intervention group, people with a lower level of drinking in negative situations reduced their bias more than people with a higher level of drinking in negative situations (Figure 6). Additionally, a longer duration of dependency (*β*=–10.92; *t*_36_=–2.54; *P*=.02) and a higher IDS “positive situations” score (*β*=–20.44; *t*_36_=–2.63; *P*=.01) were associated with a larger decrease in bias.

**Table 6:** Effect estimates for the change in the alcohol approach bias (the bias on the second examination day [T2] minus the bias on the first examination day [T1]; n=46; F9,36=2.15; *P*=.05; *R*²=0.35; adjusted *R*²=0.19).

Variables	Regression coefficient (SE)	*t* value	*P* value
Intercept	844.63 (322.31)	2.62	.01
Male	–82.17 (107.06)	–0.77	.45
Duration of dependency	–10.92 (4.30)	–2.54	.02
PSS^a^ at T1	–6.94 (8.68)	–0.80	.43
STAI^b^ State at T1	–7.27 (5.65)	–1.29	.21
STAI Trait at T1	9.42 (6.44)	1.46	.15
IDS^c^ (drinking in positive situations)	–20.44 (7.76)	–2.63	.01
IDS (drinking in negative situations)	–13.48 (14.22)	–0.95	.35
Training	–580.81 (221.94)	–2.62	.01
IDS (drinking in negative situations) × training	43.98 (15.86)	2.77	.009

^a^PSS: Perceived Stress Scale.

^b^STAI: State-Trait Anxiety Inventory.

^c^IDS: Inventory of Drinking Situations.

## Discussion

### Overview

The statistical analysis showed a general reduction in craving, alcohol approach bias, and alcohol attentional bias across the study period. We did not find any significant differences between the control and the intervention groups using repeated measures ANOVA. However, considering several factors such as gender, duration of dependency, stress, and anxiety, which could influence the outcome of the intervention, we were able to observe positive effects of the SALIENCE training. Hence, the intervention group showed better results in reducing craving and alcohol approach bias. Furthermore, we identified that the drinking motivation especially influenced the training outcome. Craving and alcohol approach bias showed a significantly greater reduction in participants consuming less alcohol in negative situations than in participants consuming more alcohol in negative situations.

### Effects of Treatment

Participants in both the control and intervention groups demonstrated a significant reduction in all craving scales over the study period. This indicates the success of the extended detoxification treatment that all participants received. However, the control and intervention groups did not differ significantly in the extent of craving reduction. Therefore, the hypothesis that the SALIENCE training, as an add-on to the usual therapy, provided additional benefit in reducing craving could not be confirmed. Similar results were found for attention, alcohol approach bias, and alcohol attentional bias.

Several reasons are conceivable for this null finding. The prolonged detoxification treatment took up a large portion of the participants’ therapy time. Therefore, craving, attention, alcohol approach bias, and alcohol attentional bias might have been more affected by the detoxification treatment than by the SALIENCE training, which was conducted only 6 times. Also, there could be a general training effect, such as improved performance on cognitive tasks with each repetition. Overall, these factors could mask the potential positive effects of the SALIENCE training.

### Factors Influencing Treatment Outcome

We observed that the amount of alcohol consumption in negative situations (measured with the IDS Negative Situations Scale) moderated the effect of the SALIENCE intervention. The less the participants in the intervention group drank alcohol in negative situations, the better the results were in terms of reducing craving for alcohol and bias toward alcohol compared to participants who drank more in negative situations. In other words, participants who did not drink alcohol due to negative affect benefited from a positive effect of the SALIENCE training by a greater decrease in craving and cognitive bias than without the intervention. These results underline that negative affect correlates positively with the strength of craving and reduces the success of therapy by causing relapses [59,60]. Craving thus occurs, especially in situations in which negative emotions arise and the addictive substance cannot be consumed, such as during addiction treatment [59].

Our results demonstrate the importance of individual therapy that considers the type of drinking behavior. While participants who do not drink out of negative affect clearly benefit from SALIENCE training, participants who tend to cope with negative emotions by consuming alcohol are more likely to show craving and cognitive bias. The greater effect of the training on participants with low scores in the IDS Negative Situations Scale may also be because the different scenarios of the training mainly depict positive and social situations, especially the risky situations of people who consume because positive rather than negative effects are simulated. People who drink out of negative affect, on the other hand, are barely confronted with their individual situations of high risk of consuming alcohol in SALIENCE training. However, new scenarios and situations can be added to the SALIENCE program, and the effectiveness of the application can be enhanced by providing more customized high-risk situations for each participant. In addition, the therapy can be supplemented with techniques that improve mood in the aforementioned patients, as an improvement in mood correlates positively with the success of the therapy [10]. Patients who drink because of negative effects can also benefit more from mindfulness-based therapy [61].

### Limitations

One limitation of this study is that the groups were not homogeneous in terms of age and duration of dependency. The intervention group was younger and had a shorter duration of dependency than the control group. This might have influenced the comparability of the groups. Collecting data from a larger sample or a group matching technique could have counteracted it.

A more discriminating assessment of craving would have been advantageous. For instance, the Penn Alcohol Craving Scale (PACS) could have been used in addition to the OCDS [62]. Unlike the multidimensional OCDS, the PACS is a single-factor instrument. The OCDS also addresses other aspects of alcohol dependence, such as obsessive thoughts about alcohol and the compulsion to drink. The PACS enables us to measure craving without further facets influencing addiction [62].

Also, more training sessions could possibly have resulted in greater success of the intervention; however, it was found that 6 repetitions of CBMT already had a significant effect on craving [63]. Nevertheless, most patients would benefit more from continuing the training.

In addition, the training sessions were not adapted to the individual high-risk situations. In the future, participants should be able to add individual scenarios that allow highly individualized therapy. Additionally, an enhancement of virtual reality by addressing different senses could allow the CET to appear even more realistic. For example, the virtual presentation of visual stimuli could be supplemented by alcohol-associated acoustic stimuli (eg, clinking of glasses, music, and people chatting) and olfactory stimuli (eg, smell of alcohol).

Furthermore, it should be noted that the neuropsychological test battery is also a form of CBMT, and consequently, the control group also benefited from CBMT to a certain extent [53].

### Conclusions

We investigated the effectiveness of SALIENCE computer-based training by performing a randomized controlled trial. A reduction in craving, alcohol approach bias, and alcohol attentional bias was observed in both groups. Taking into account factors that could modulate the therapeutic success of addiction, we found that SALIENCE training has a positive influence on treating AUD. Craving and alcohol approach bias could be reduced more with the implementation of the training than with standard therapy alone. It was noticeable that individuals with AUD who barely consume alcohol in negative situations benefit from the training and have a better outcome than without the training. In particular, the parameters of craving and alcohol approach bias could be significantly reduced due to the intervention in this subset of patients.

## Appendix

Multimedia Appendix 1 CONSORT-EHEALTH v1.6.

Multimedia Appendix 2 Sociodemographic variables and questionnaire scores at baseline, Alcohol Stroop Task results, and Dot Probe Task results.

## Abbreviations

AAT: Approach Avoidance Task
ADHD-SB: Attention-Deficit/Hyperactivity Disorder Self-Assessment Scale
ADS: Alcohol Dependence Scale
AUD: alcohol use disorder
BAI: Beck Anxiety Inventory
BDI: Beck Depression Inventory
CAS-A: Craving Automated Scale for Alcohol
CBMT: Cognitive Bias Modification Therapy
CET: cue-exposure therapy
CIWA‑Ar: Clinical Institute Withdrawal Assessment for Alcohol
CONSORT: Consolidated Standards of Reporting Trials
DSM-5: Diagnostic and Statistical Manual of Mental Disorders, 5th edition
IDS: Inventory of Drinking Situations
OCDS: Obsessive Compulsive Drinking Scale
PACS: Penn Alcohol Craving Scale
PANAS: Positive and Negative Affect Schedule
PSS: Perceived Stress Scale
SALIENCE: Stop Alcohol in Everyday Life-New Choices and Evaluations
SRHI: Self-Report Habit Index
STAI: State-Trait Anxiety Inventory
STDS: State-Trait Depression Scale
T0: screening day
T1: first examination day
T2: second examination day
