# Supplementary material for: Effectiveness of Reducing Craving in Alcohol Use Disorder Using a Serious Game (SALIENCE): Randomized Controlled Trial
Source: JMIR Form Res. 2023 Nov 7;7:e42194. doi: 10.2196/42194 (PMC10664013; doi:10.2196/42194)
Supplement: Multimedia Appendix 2 [file formative_v7i1e42194_app2.docx]

# Effectiveness of the serious game SALIENCE on craving and approach avoidance bias in alcohol use disorder: a randomized controlled trial

## Supplementary material

**Table S1.** Sociodemographic variables and questionnaire scores at baseline.

|  | Intervention group (*n* = 27) | Control  group (*n* = 22) | Test statistic**^h^** | *p* |
| --- | --- | --- | --- | --- |
|  |  |  |  |  |
| **Age in years, m (SD)** |  |  |  |  |
|  | 40.56 (11.59) | 48.32 (12.18) | t (47) = -2.27 | .028 |
| **Female, n (%)** |  |  |  |  |
|  | 6 (22) | 11 (50) | χ(1) = 2.99^i^ | .084 |
| **Married, n (%)** |  |  |  |  |
|  | 4 (15) | 5 (23) | χ(3) = 2.44 | .490 |
| **Duration of dependence in years, m (SD)** |  |  |  |  |
|  | 25.70 (8.66) | 36.09 (13.87) | t(34) = -3.06 | .004 |
| **Previous inpatient detoxification, n (%)** |  |  |  |  |
|  | 13 (48) | 10 (45) | χ(1) = 0.00 | 1.000 |
| **Psychological comorbidities, n (%)** |  |  |  |  |
|  | 15 (56) | 12 (54) | χ(1) = 0.0 | 1.000 |
| **ADS^a^ sum score, m (SD)** |  |  |  |  |
|  | 18.69 (7.70) | 16.14 (9.25) | t(46) = 1.03 | .310 |
| **IDS100^b^ positive situations, m (SD)** |  |  |  |  |
|  | 12.99 (7.07) | 12.11 (7.29) | t(46) = 0.42 | .676 |
| **IDS100 negative situations, m (SD)** |  |  |  |  |
|  | 11.88 (5.03) | 11.12 (6.29) | t(46) = 0.46 | .651 |
| **BDI^c^ sum score, m (SD)** |  |  |  |  |
|  | 18.33 (11.82) | 13.95 (12.52) | t(47) = 1.25 | .219 |
| **BAI^d^ sum score, m (SD)** |  |  |  |  |
|  | 16.53 (12.77) | 13.18 (14.51) | t(46) = 0.84 | .405 |
| **PANAS^e^ positive affect trait, m (SD)** |  |  |  |  |
|  | 25.15 (7.78) | 26.09 (10.93) | t(47) = -0.34 | .735 |
| **PANAS negative affect trait, m (SD)** |  |  |  |  |
|  | 27.52 (7.03) | 22.68 (9.76) | t(47) = 1.95 | .059 |
| **ADHD^f^ sum score, m (SD)** |  |  |  |  |
|  | 18.11 (9.23) | 14.73 (11.70) | t(47) = 1.11 | .276 |
| **F90^g^ consumption of alcohol in grams, m (SD)** |  |  |  |  |
|  | 9573.31 (7979.91) | 11395.68 (7827.36) | t(46) = -0.80 | .430 |
| **F90 drinks per day, m (SD)** |  |  |  |  |
|  | 8.86 (7.39) | 10.55 (7.25) | t(46) = -0.80 | .430 |
| **F90 drinks per drinking day, m (SD)** |  |  |  |  |
|  | 15.75 (9.11) | 15.79 (9.23) | t(46) = -0.02 | .987 |
| **F90 drinks per heavy drinking day, m (SD)** |  |  |  |  |
|  | 25.91 (29.80) | 18.88 (14.05) | t(46) = 1.20 | .292 |
| **F90 percent days abstinent, m (SD)** |  |  |  |  |
|  | 44.10 (28.36) | 33.94 (23.84) | t(46) = 1.35 | .184 |
| **F90 percent drinking days, M (SD)** |  |  |  |  |
|  | 55.90 (28.36) | 66.57 (23.45) | t(46) = -1.43 | .161 |
| **F90 percent heavy drinking days, m (SD)** |  |  |  |  |
|  | 49.83 (31.42) | 62.37 (26.59) | t(46) = -1.50 | .141 |

^a^ADS: Alcohol Dependence Scale

^b^IDS: Inventory of Drinking Situations

^c^BDI: Beck Depression Inventory

^d^BAI: Beck Anxiety Inventory ^e^PANAS: Positive and Negative Affect Schedule ^f^ADHD: Attention Deficit Hyperactivity Disorder ^g^F90: Form 90 ^h^We performed the Welch’s t-test. Some participants had missing values which lead to different degrees of freedom.

^I^ Pearson’s Chi-squared test with Yates’ continuity correction

### Supplementary material Alcohol Stroop Task

In the following, the categories of the Alcohol Stroop Task are named as follows: "Category 1" = "letters 1", "Category 2" = "household", "Category 3" = "no alcohol”, "Category 4" = "alcohol” and "Category 5" = "letters 2”.

In addition to the mean reaction times of the individual categories, the difference of the average reaction times of the categories "alcohol" minus "household" was calculated. Using repeated measures ANOVAs we found that the mean reaction times in the Alcohol Stroop Task of the associated expressions of the categories "letters 1" [F (1.00, 47.00) = 4.15; p =.047], "household" [F (1.00, 46.00) = 5.74; p =.021], "no alcohol" [F (1.00, 47.00) = 9.76; p =.003] and "alcohol" [F (1.00, 47.00) = 6.46; p =.014] decreased significantly over the study period. Apart from that, no relevant changes were calculated with the main factor of time. There were also no relevant time-group interactions (Table 1).

**Table S2.** Alcohol stroop task .

|  | Intervention group (n=27) | | Control group  (n=22) | | Effect of time test | Time x group interaction test |
| --- | --- | --- | --- | --- | --- | --- |
|  | T1^a^ | T2^b^ | T1 | T2 |  |  |
|  |  |  |  |  |  |  |
| **Mean reaction times in ms^c^ letters 1, m (SD)** | 768.34 (123.40) | 726.68 (96.95) | 917,04 (193.46) | 884.49 (194.27) | F (1.00, 47.00) = 4.15; p =.047 | F (1.00, 47.00) = 0.06; p =.804 |
|  |  |  |  |  |  |  |
| **Mean reaction times in ms household, m (SD), n=49/21** |  |  |  |  |  |  |
|  | 771.80 (121.08) | 738.43 (112.04) | 923.14 (193.07) | 875.11 (196.57) | F (1.00, 46.00) = 5.74; p =.021 | F (1.00, 47.00) = 0.19; p =.668 |
| **Mean reaction times in ms no alcohol, m (SD)** |  |  |  |  |  |  |
|  | 777.58 (119.60) | 726.16 (95.81) | 923.73 (191.09) | 875.29 (191.09) | F (1.00, 47.00) = 9.76; p =.003 | F (1.00, 47.00) = 0.01; p =.975 |
| **Mean reaction times in ms alcohol, m (SD)** |  |  |  |  |  |  |
|  | 772.25 (125.88) | 736.13 (104.76) | 928.53 (182.77) | 885.85 (185.22) | F (1.00, 47.00) = 6.46; p =.014 | F (1.00, 47.00) = 0.05; p =.833 |
| **Mean reaction times in ms letters 2, m (SD)** |  |  |  |  |  |  |
|  | 761.61 (119.51) | 725.31 (113.68) | 910.13 (199.11) | 879.11 (185.11) | F (1.00, 47.00) = 3.23; p =.078 | F (1.00, 47.00) = 0.02; p =.888 |
| **Number of correct answers letters 1, m (SD)** |  |  |  |  |  |  |
|  | 77.19 (4.58) | 75.81 (8.90) | 77.73 (2.10) | 77.27 (5.35) | F (1.00, 47.00) = 0.81; p =.374 | F (1.00, 47.00) = 0.20; p =.654 |
| **Number of correct answers household, m (SD)** |  |  |  |  |  |  |
|  | 77.81 (3.77) | 75.93 (8.50) | 77.27 (2.76) | 77.00 (5.33) | F (1.00, 47.00) = 1.14; p =.291 | F (1.00, 47.00) = 0.64; p =.429 |
| **Number of correct answers no alcohol, m (SD)** |  |  |  |  |  |  |
|  | 77.15 (4.01) | 76.04 (8.89) | 78.05 (2.06) | 78.00 (3.39) | F (1.00, 47.00) = 0.39; p =.534 | F (1.00, 47.00) = 0.33; p =.567 |
| **Number of correct answers alcohol, m (SD)** |  |  |  |  |  |  |
|  | 77.22 (4.04) | 75.23 (4.84) | 77.82 (1.89) | 77.23 (4.84) | F (1.00, 47.00) = 1.29; p =.262 | F (1.00, 47.00) = 0.23; p =.636 |
| **Number of correct answers letters 2, m (SD)** |  |  |  |  |  |  |
|  | 38.41 (2.21) | 37.70 (4.48) | 38.82 (1.40) | 38.68 (3.71) | F (1.00, 47.00) = 0.76; p =.389 | F (1.00, 47.00) = 0.35; p =.560 |
| **Mean reaction times in ms alcohol - household, m (SD)** |  |  |  |  |  |  |
|  | 0.46 (43.98) | -2.30 (37.51) | -4.24 (34.39) | -2.60 (37.37) | F (1.00, 47.00) = 0.01; p =.948 | F (1.00, 47.00) = 0.07; p =.795 |

^a^T1: first examination day

^b^T2: second examination day

^c^ms: milliseconds

### Supplementary material Dot Probe Task

**Table S3.** Dot Probe Task

|  | Intervention group (n=25) | | Control group  (n=22) | | Effect of time test | Time x group interaction test |
| --- | --- | --- | --- | --- | --- | --- |
|  | T1^a^ | T2^b^ | T1 | T2 |  |  |
|  |  |  |  |  |  |  |
| **Number of correct answers congruent, m (*SD*)** |  |  |  |  |  |  |
|  | 14.72 (4.29) | 15.86 (0.37) | 16.00 (0.00) | 15.86 (0.35) | F (1.00, 45.00) = 1.11; p =.297 | F (1.00, 45.00) = 1.82; p =.184 |
| **Number of correct answers incongruent, m (*SD*)** |  |  |  |  |  |  |
|  | 15.80 (0.50) | 16.00 (0.00) | 15.64 (1.71) | 15.77 (0.69) | F (1.00, 45.00) = 1.83; p =.183 | F (1.00, 45.00) = 0.07; p =.799 |
| **mean reaction time in ms^c^ congruent, m (SD), n=24/22** |  |  |  |  |  |  |
|  | 479.16 (146.69) | 500.77 (92.55) | 595.61 (154.33) | 571.77 (184.43) | F (1.00, 45.00) = 0.01; p =.955 | F (1.00, 45.00) = 1.24; p =.272 |
| **mean reaction time in ms incongruent, m (SD),** |  |  |  |  |  |  |
|  | 495.09 (112.52) | 506.95 (100.64) | 620.44 (163.69) | 582.95 (147.20) | F (1.00, 45.00) = 0.45; p =.497 | F (1.00, 45.00) = 1.65; p =.205 |
| **Dot Probe Score (mean reaction times incongruent - congruent), m (SD), n=24/22** |  |  |  |  |  |  |
|  | 34.90 (167.41) | -1.42 (26.73) | 24.83 (56.95) | 11.18 (26.12) | F (1.00, 45.00) = 1.78; p =.189 | F (1.00, 45.00) = 0.37, p =.548 |

^a^T1: first examination day

^b^T2: second examination day

^c^ms: milliseconds
